# Predictors of Lesion Cavitation After Recent Small Subcortical Stroke

**DOI:** 10.1007/s12975-019-00741-8

**Published:** 2019-11-08

**Authors:** Thomas Gattringer, Maria Valdes Hernandez, Anna Heye, Paul A Armitage, Stephen Makin, Francesca Chappell, Daniela Pinter, Fergus Doubal, Christian Enzinger, Franz Fazekas, Joanna M. Wardlaw

**Affiliations:** 1 Department of Neurology, Medical University of Graz, Graz, Austria; 2 Centre for Clinical Brain Sciences, The University of Edinburgh, Chancellor’s Building, 49 Little France Crescent, Edinburgh, EH16 4SB UK; 3 UK Dementia Research Institute, The University of Edinburgh, Edinburgh, UK; 4 Department of Infection, Immunity and Cardiovascular Disease, University of Sheffield, Sheffield, UK; 5 Academic Section of Geriatric Medicine, Institute of Cardiovascular and Medical Sciences, University of Glasgow, Glasgow, UK

**Keywords:** Lacunar stroke, Recent small subcortical infarction, MRI, Blood-brain barrier, Diffusion tensor imaging

## Abstract

Morphologic evolution of recent small subcortical infarcts (RSSI) ranges from lesion disappearance to lacune formation and the reasons for this variability are still poorly understood. We hypothesized that diffusion tensor imaging (DTI) and blood-brain-barrier (BBB) abnormalities early on can predict tissue damage 1 year after an RSSI. We studied prospectively recruited patients with a symptomatic MRI-defined RSSI who underwent baseline and two pre-specified MRI examinations at 1–3-month and 1-year post-stroke. We defined the extent of long-term tissue destruction, termed *cavitation index*, as the ratio of the 1-year T1-weighted cavity volume to the baseline RSSI volume on FLAIR. We calculated fractional anisotropy and mean diffusivity (MD) of the RSSI and normal-appearing white matter, and BBB leakage in different tissues on dynamic contrast-enhanced MRI. Amongst 60 patients, at 1-year post-stroke, 44 patients showed some degree of RSSI cavitation on FLAIR, increasing to 50 on T2- and 56 on T1-weighted high-resolution scans, with a median cavitation index of 7% (range, 1–36%). Demographic, clinical, and cerebral small vessel disease features were not associated with the cavitation index. While lower baseline MD of the RSSI (*r*_s_ = − 0.371; *p* = 0.004) and more contrast leakage into CSF (*r*_s_ = 0.347; *p* = 0.007) were associated with the cavitation index in univariable analysis, only BBB leakage in CSF remained independently associated with cavitation (beta = 0.315, *p* = 0.046). Increased BBB leakage into CSF may indicate worse endothelial dysfunction and increased risk of tissue destruction post RSSI. Although cavitation was common, it only affected a small proportion of the original RSSI.

**Electronic supplementary material:**

The online version of this article (10.1007/s12975-019-00741-8) contains supplementary material, which is available to authorized users.

## Introduction

Recent small subcortical infarcts (RSSIs) are the neuroimaging correlate of acute lacunar strokes, which make up to 25% of all ischemic strokes [1, 2]. RSSIs have different morphological fates. Besides the classical evolution to a lacunar cavity, they can also turn to a more unspecific lesion (i.e., a non-cavitated hyperintensity on T2- or FLAIR-weighted MRI scans) and some vanish after several weeks or months [3–7]. Prior studies have mostly considered FLAIR- and/or T2-weighted MRI sequences to rate the final tissue outcome of an RSSI. However, isotropic, high-resolution T1 sequences increase the sensitivity of detecting small lacunar lesions following an RSSI, yielding some degree of cavitation in > 90% [3, 5].

The causes and mechanisms that determine the morphologic evolution of an RSSI are incompletely understood. Factors leading to more or less tissue destruction would not only be relevant regarding a more refined understanding of the pathophysiology of cerebral small vessel disease (CSVD) but could also be clinically relevant (e.g., enhance understanding of the risk of cognitive impairment), since cavitation is likely to indicate worse tissue destruction than with non-cavitated or vanishing lesions, and apply to symptomatic and to asymptomatic acute small subcortical lesions [8] that contribute to accumulating brain damage.

Few studies have assessed the capacity of advanced MRI techniques to predict tissue destruction following an RSSI. A promising candidate is diffusion tensor imaging (DTI) by indicating the initial severity of ischemia and of microstructural (dis)integrity [9]. Another candidate is dynamic contrast-enhanced (DCE) MRI as increased general blood-brain barrier (BBB) leakage in subcortical tissues and into CSF has been seen in patients with more severe microvascular brain changes [10, 11], and could also be associated with increased focal morphological damage following an RSSI.

We hypothesized that (1) more severe DTI abnormalities of the RSSI at baseline and (2) greater BBB leakage 1–3-month post-stroke on DCE MRI would be associated with more severe focal tissue destruction 1 year after an RSSI. We used a high-resolution T1 sequence to increase the sensitivity for detecting cavity formation and quantified the amount of “total tissue destruction” as the ratio of the T1 cavity volume at year 1 and the FLAIR infarct volume at baseline, which we termed the *cavitation index*.

## Methods

Data that support the findings of this study are available from the corresponding author upon reasonable request.

### Study Participants

RSSI patients were selected from the prospective observational “Mild Stroke Study 2” (MSS2) that was conducted between May 1, 2010, and May 31, 2013. In brief, MSS2 prospectively recruited ischemic stroke patients who presented to the Lothian Stroke Service in Edinburgh, UK, with symptoms of mild stroke (i.e., National Institutes of Health Stroke Scale score [NIHSS] ≤ 7) including lacunar and cortical subtypes. All patients had a thorough clinical examination by dedicated stroke physicians and detailed diagnostic workup including brain MRI at presentation. All patients were also invited for a second brain MRI scan at 1–3 month(s) after stroke to assess BBB leakage on dynamic contrast-enhanced (DCE) imaging, and for a third MRI at 1 year after stroke to assess changes in vascular brain lesions. More details of the MSS2 study have been published previously [12, 13].

For the present study, we considered all MSS2 study participants with the following inclusion criteria:

- Stroke symptoms with a compatible MRI-defined RSSI < 20 mm in axial diameter and located in the supply area of a small perforating brain artery,
- Availability of 1–3-month MRI including DCE scans to assess BBB leakage,
- Availability of 1-year MRI for rating of RSSI lesion change.

### Magnetic Resonance Imaging Acquisition

All three MRI examinations were performed on the same scanner (1.5 T Signa HDxt, General Electric, Milwaukee, WI), the stability of which was maintained and monitored under a tight quality assurance program. Diagnostic MRI at presentation included diffusion tensor imaging (DTI), FLAIR-, T2-, T2*-, and T1-weighted volume sequences (all 1 mm slice gap). The same sequences were used at 1 year. Detailed technical information on the MRI protocol is provided elsewhere [12].

DCE MRI to assess BBB leakage was performed at 1–3 month(s) after the index stroke to minimize the effect of the acute stroke lesion on BBB. After two 3D fast spoiled gradient-echo acquisitions (flip angles 2 and 12°) for pre-contrast T1 (T10) maps, gadoterate meglumine (Gd-DOTA, DOTAREM; Guerbet, Paris, France) 0.2 mL/kg (i.e., 0.1 mmol/kg body weight) was injected at 2 mL/s intravenously via an injection pump and then the 3D T1-weighted sequence was repeated sequentially 20 times for 24 min, using long acquisition times to detect subtle BBB leak [13–15].

### Image Analysis

All image analysis was blinded to patient characteristics, clinical outcomes, and BBB and DTI measures. Additionally, different components of the image analysis were performed by different individuals to maintain blinding.

#### MRI Assessment at Baseline

All baseline scans were analyzed for the RSSI and chronic tissue changes characteristic for cerebral small vessel disease (CSVD). White matter hyperintensities (WMH), lacunes of presumed vascular origin, microbleeds, and enlarged perivascular spaces (PVS) were identified according to the STRIVE criteria [2] by an experienced neuroradiologist (JMW). WMH were rated according to the Fazekas scale [16]; superficial and deep atrophy and perivascular spaces (PVS) were graded according to previously validated scales [17, 18].

#### Rating and Quantitative Assessment of RSSI Evolution

Tissue outcome of the RSSI at the 1–3-month post-stroke and 1 year post-stroke on MRI was rated by an experienced MRI reader (TG) using co-registered FLAIR-, T2-, and T1-weighted sequences. A lacunar cavity was defined as a lesion with CSF signal intensity on respective MRI scans.

The cavitation index= $\frac{\mathrm{Cavity}\ \mathrm{volume}\ \mathrm{on}\ \mathrm{T}1\ \mathrm{at}\ 1\ \mathrm{year}}{\mathrm{RSSI}\ \mathrm{volume}\ \mathrm{on}\ \mathrm{FLAIR}\ \mathrm{at}\ \mathrm{baseline}}$ was defined as the primary tissue outcome variable. A similar approach has previously been used in studies in multiple sclerosis to determine the “black hole ratio” (i.e., the proportion of T2-weighted hyperintense lesions that later developed into T1-weighted hypointense “black holes”) [19, 20].

#### Magnetic Resonance Image Processing

Boundaries of the RSSI on the original baseline FLAIR images were delineated on all slices on which it was visible, using the software Mango (http://ric.uthscsa.edu/mango/) and guided by the initial DWI sequence. A “control” region symmetric to the RSSI in the normal-appearing tissue of the contralateral hemisphere was also drawn, being careful to avoid areas of coexisting morphologic damage including WMH. At 1-year follow-up MRI, T1 hypointensity of the index RSSI was also manually outlined. Identification of these lesions was guided by concurrent MRI scans. We were careful to avoid misclassifying a T1 hypointensity as a PVS by looking at baseline MRI scans (including co-registration). For inter-rater reliability measurements, cavitation volume at 1 year on T1 as the main tissue outcome parameter was also outlined and calculated by a second rater (MVH) with different professional background (see supplementary material).

#### Diffusion Analysis

Parametric maps of fractional anisotropy (FA) and mean diffusivity (MD) maps were generated from the DTI acquisition as described elsewhere [21]. All regions of interest (ROIs) were mapped from the structural to the diffusion space using NiftyReg (http://sourceforge.net/projects/niftyreg/) applied using TractoR software (http://www.tractor-mri.org.uk/diffusion-processing). In the registration process, we used trilinear interpolation, preferred in reconstruction algorithms [22], given the precision required in the space manipulation of such small ROIs to avoid shape and size distortion. The resultant ROIs were binarized and, then, multiplied by the FA and MD maps to calculate the median values of FA and MD in each ROI. To evaluate the effect of partial volume effects of the RSSI and “control” ROIs with CSF, the areas of intersection of these ROIs with a binary mask of CSF in the diffusion space, generated from a previous study [21], were removed and median values were also obtained from these “noCSF” resultant ROIs. MD and FA values of the RSSI were expressed relative to the contralateral “control” side.

#### DCE MRI Analysis

The area under the curve (AUC) of the signal enhancement curves *E*(*t*) from *t* = 0 (administration of contrast agent) to *t* = 24 min, which is a semi-quantitative measure of contrast uptake in tissue, was used as the estimate for BBB leakage. It was measured separately in CSF, normal-appearing white and deep gray matter, WMH, and RSSI, which were delineated semiautomatically on co-registered images by an image analyst (MVH) using the in-house developed software MCMxxxVI (https://sourceforge.net/projects/bric1936/). All segmented tissue masks were manually checked and edited as necessary. In order to correct for inter-subject variability of the vascular input, the AUC of the respective regions of interest was normalized to the AUC of the vascular signal enhancement curve in the superior sagittal sinus yielding the blood-normalized AUC, as previously reported [23, 24].

### Statistical Analysis

Statistical analyses were performed using the Statistical Package of the Social Sciences (IBM SPSS Statistics 23). The level of significance was set at *p* < 0.05. The Kolmogorov-Smirnov test and visual inspection of histograms assessed normality of data distribution. Groups were compared by the chi-square test (for nominal data), Students *t* test (for continuous, normally distributed variables), or the Mann–Whitney *U* test (for non-normally distributed variables). Patients were dichotomized according to median split of the primary outcome variable *cavitation index*. Correlation analysis was performed using Spearman correlation.

After logarithmic transformation of the target variable *cavitation index*, we also performed two linear regression analyses based on our pre-specified hypotheses and the results from univariable analyses. Covariates for the two models were added on the basis of biological plausibility and a literature review [3, 4].

#### Model 1: Cavitation Index and RSSI MD

The first multivariable model for the dependent variable *cavitation index* included patient age, the time interval from stroke symptom onset to baseline MRI, WMH lesion load (sum of deep and periventricular WMH scores according to the Fazekas scale), RSSI size at baseline, and the median MD value of the RSSI at baseline.

#### Model 2: Cavitation Index and BBB Leak in the CSF

The second multivariable model for the target variable *cavitation index* comprised patient age, the time period from stroke symptom onset to DCE MRI at 1–3 months, WMH lesion load, RSSI size, and BBB leakage in the CSF at 1–3-month post-stroke.

## Results

We identified 60 stroke patients (mean age 65.1 ± 10.6 years, 65% male) with a single symptomatic RSSI who fulfilled the inclusion criteria. DCE MRI data were unavailable in one patient, therefore 60 patients contributed to cavitation and DTI, and 59 to BBB analysis. Further clinical and neuroimaging characteristics of the study cohort are shown in Table 1.

**Table 1:** Baseline clinical and MRI characteristics of the total cohort and comparison of subgroups according to the median split of the *cavitation index*

Variables	Total cohort, *n* = 60	Cavitation index ≤ 7%	Cavitation index > 7%	*p* value
		*N* = 29	*N* = 31	
Age in years, mean (± SD)	65.1 (10.6)	66.1 (9.8)	64.1 (11.4)	0.47
Male sex, *n* (%)	39 (65)	21 (72)	18 (58)	0.65
NIHSS, median (range)	1 (0–7)	1 (0–5)	1 (0–7)	0.20
Modified Rankin Scale, median (IQR)	1 (2)	1 (1)	2 (2)	0.170
Vascular risk factors, *n* (%)				
Hypertension	46 (77)	26 (84)	20 (69)	0.17
Diabetes	6 (10)	4 (13)	2 (7)	0.44
Hyperlipidemia	39 (65)	20 (65)	19 (66)	0.94
Smoking	24 (40)	12 (39)	12 (41)	0.83
Baseline MRI characteristics				
Symptom onset to baseline MRI in days, median (IQR)	4 (4)	4 (5)	4 (3.5)	0.79
Symptom onset to FU 1 MRI in days, median (IQR)	45 (29)	47.5 (28.25)	41 (31)	0.33
Symptom onset to FU 2 MRI in days, median (IQR)	386 (53)	392 (65.25)	384 (49)	0.41
Maximal axial RSSI diameter in mm, median (IQR)	12 (7)	12 (6)	10.5 (7.6)	0.29
RSSI location, *n* (%)				0.42
Basal ganglia/internal capsule	15 (25)	7 (23)	8 (28)	
Centrum semiovale	25 (42)	15 (48)	10 (35)	
Thalamus	14 (23)	5 (16)	9 (31)	
Brainstem	6 (10)	4 (13)	2 (7)	
Fazekas WMH sum score* (0–6), median (IQR)	3 (3)	3.5 (1–6)	3 (2–6)	0.16
Lacunar infarcts ≥ 1, *n* (%)	30 (50)	16 (52)	14 (48)	0.8
Microbleeds ≥ 1, *n* (%)	17 (28)	12 (40)	5 (17)	0.054
PVS in basal ganglia (0–4), median (IQR)	2 (2)	2 (1–4)	2 (1–4)	0.97
PVS in centrum semiovale (0–4), median (IQR)	2 (2)	2 (0–4)	2 (1–4)	0.73
Deep atrophy score (0–3), median (IQR)	1 (1)	1 (0–3)	1 (0–3)	0.047
Superficial atrophy score (0–3), median (IQR)	1 (1)	1 (0–3)	1 (0–2)	0.56
Cavitation index at 1 year, median (range)	0.071 (0.01–0.36)	0.045 (0.01–0.07)	0.116 (0.08–0.36)	< 0.001
Modified Rankin Scale at 1 year, median (IQR)	1 (1)	1 (2)	1 (1)	0.476
ACE-R [37] at 1 year, median (IQR)#	90 (13)	91.5 (12)	88.5 (15)	0.52

*Sum of periventricular (0–3) and deep (0–3) WMH scores

#Available in 42 patients (*n* = 22 with cavitation index ≤ 7%; *n* = 20 with cavitation index > 7%)

*SD*, standard deviation; *NIHSS*, National Institutes of Health Stroke Scale; *FU*, follow-up; *RSSI*, recent small subcortical infarct; *PVS*, perivascular spaces; *IQR*, interquartile range; *ACE-R*, Addenbrooke’s Cognitive Examination Revised

**Fig. 1:**
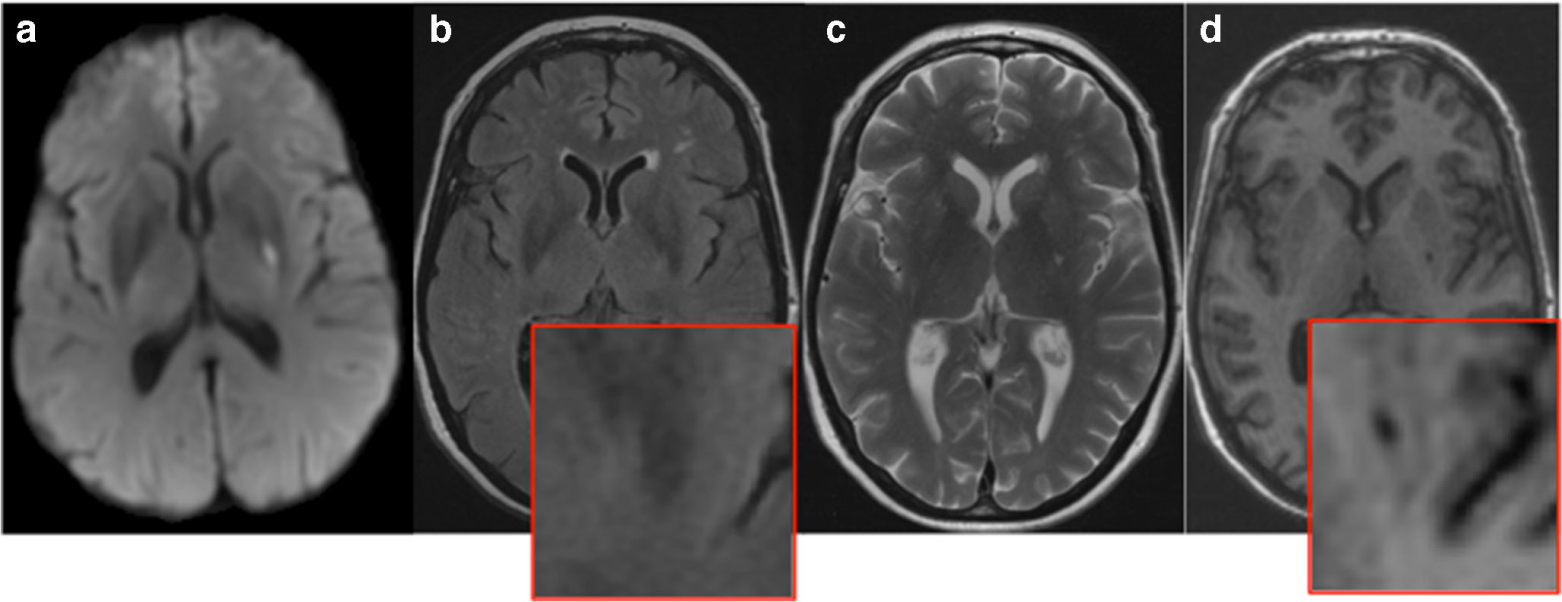
Putaminal RSSI on DWI MRI sequences 2 days after stroke onset (A). At 1-year post-stroke, there is no obvious lesion on FLAIR-weighted MRI (B, see also magnification in inset), whereas T2- (C) and T1-weighted (D) scans display a small cavity

**Fig. 2:**
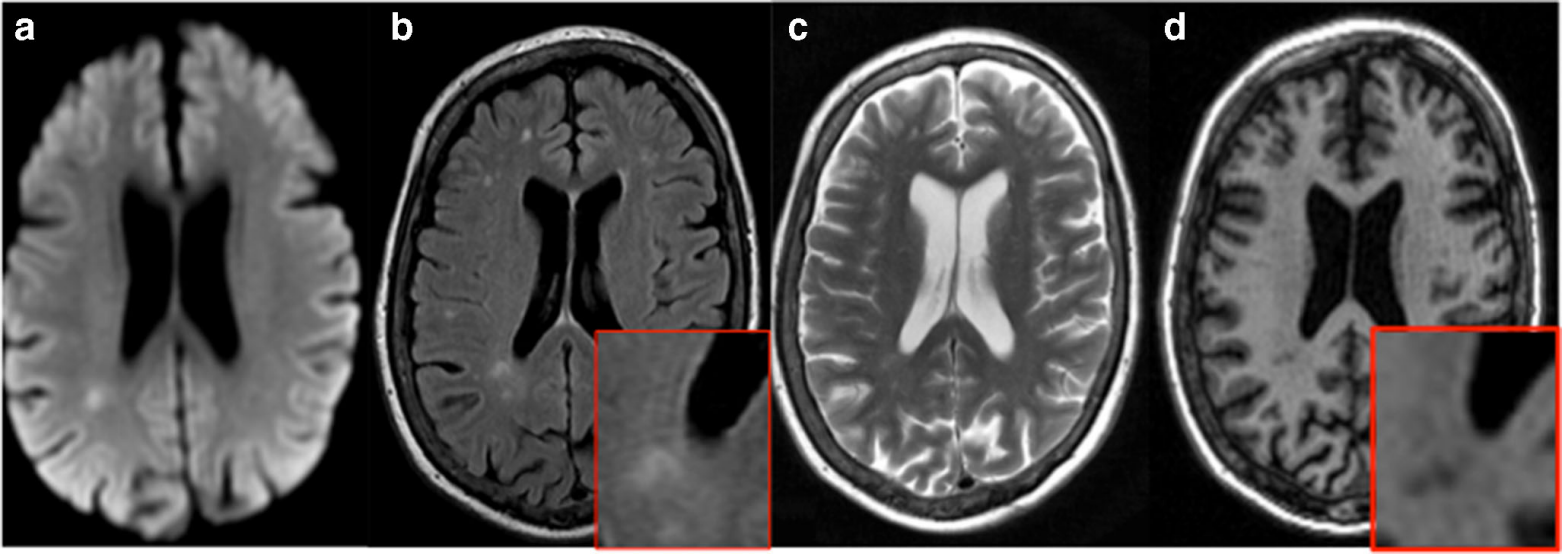
Example of an RSSI with subtle cavitation at 1 year. Index RSSI on DWI MRI (A). At 1-year post-stroke, there is no indication for cavitation on FLAIR- or T2-weighted scans (B and C). Also, T1 shows only subtle cavity formation, which becomes clearer after image magnification (D)

At 1-year post-stroke, 44 patients (73%) showed some evidence of cavitation on FLAIR-weighted MRI scans. This number increased to 50 (83%) when additionally considering T2-weighted scans. On high-resolution isotropic T1 scans, 56 (93%) patients had some cavitation of the index RSSI (Fig. 1). The remaining four patients also had small T1 hypointensities, which became visible after lesion magnification (Fig. 2). These T1 lesions were included in the calculation of the *cavitation index* (subsequent results did not significantly change after exclusion of these patients).

**Fig. 3:**
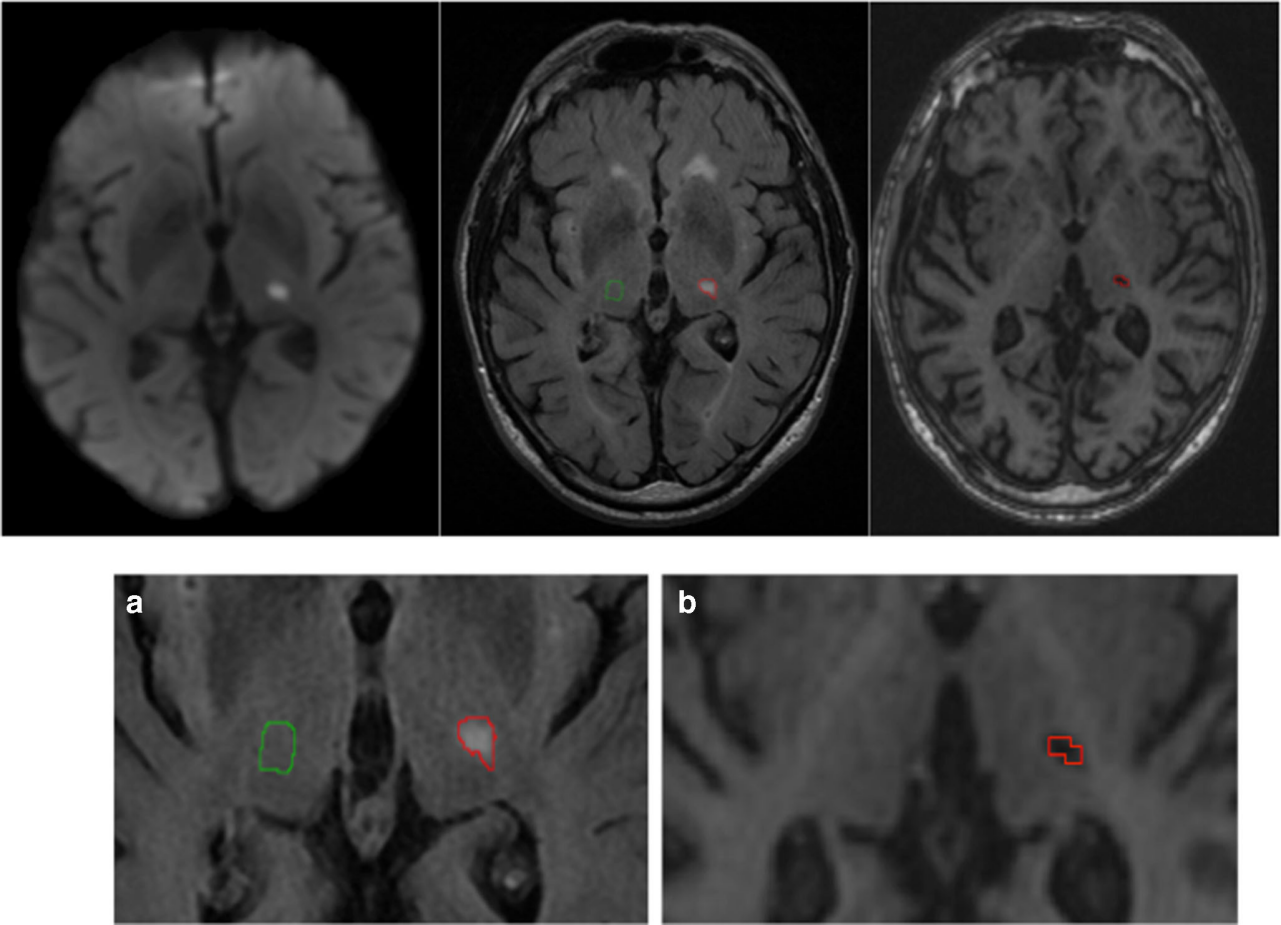
Calculation of the RSSI cavitation index. The index RSSI in the thalamus is segmented on FLAIR scans at baseline (outlined in red). The green mask illustrates the contralateral control region (image A). Image B shows the evolution of the RSSI at 1-year follow-up as a cavity on T1. The cavity was again segmented (displayed in red). The cavitation index is calculated as the ratio between the volume of the T1 cavity at 1 year and the FLAIR volume of the RSSI at baseline (= 27% in this case)

Median *cavitation index* at 1-year follow-up was 7% and ranged from 1 to 36% (median RSSI lesion volume on FLAIR at baseline 1000 mm^3^, IQR 942 mm^3^; median cavity volume at 1 year on T1 65 mm^3^, IQR 78 mm^3^). Therefore, patients were further dichotomized according to a *cavitation index* of ≤ and > 7% (median split*,* Table 1) for statistical modeling. Figure 3 shows an example of a patient with a *cavitation index* of 27%. Results from inter-rater reliability measurements are provided as online supplementary material; volumetric difference of the T1 cavity volume at 1 year between the two observers was 2%.

Cavitation status was not associated with functional neurological or cognitive outcome at 1 year (Tables 1 and 2).

**Table 2:** Bivariate correlations of clinical and MRI parameters with *cavitation index*

Variable	Spearman correlation coefficient	*p* value
Baseline variables		
Age	− 0.096	0.47
NIHSS	0.060	0.65
Time stroke symptom onset to MRI at baseline	− 0.074	0.58
Time stroke symptom onset to MRI at follow-up 1 (BBB measurements)	− 0.083	0.53
Time stroke symptom onset to MRI at follow-up 2 (rating of tissue outcome)	− 0.203	0.12
RSSI maximal diameter	− 0.142	0.28
RSSI volume	− 0.140	0.29
WMH periventricular	− 0.092	0.49
WMH deep	− 0.178	0.18
WMH sum score	− 0.137	0.30
Deep atrophy score	− 0.210	0.11
Superficial atrophy score	− 0.138	0.31
Number of microbleeds	− 0.186	0.16
Number of old lacunes	0.118	0.37
DTI parameters at baseline		
Median FA of normal-appearing white matter	− 0.001	1
Median MD of normal-appearing white matter	0.012	0.93
Median FA of RSSI (corrected for contralateral control region)	− 0.084	0.52
Median MD of RSSI (corrected for contralateral control region)	*− 0.371*	*0.004*
BBB measures at 1–3-month post-stroke*		
Normalized AUC CSF	*0.347*	*0.007*
Normalized AUC gray matter	0.218	0.10
Normalized AUC white matter	0.080	0.55
Normalized AUC white matter lesions	0.109	0.41
Normalized AUC RSSI	− 0.014	0.92
Clinical outcome at 1 year		
Modified Rankin scale	0.149	0.26
ACE-R [37] #	− 0.067	0.67

*BBB analyses were available in 59 patients (DCE data not available for 1 patient)

# Available in 42 patients

*NIHSS*, National Institutes of Health Stroke Scale; *AUC*, area under the curve; *RSSI*, recent small subcortical infarct; *DTI*, diffusion tensor imaging; *WMH*, white matter hyperintensities; *MD*, mean diffusivity; *FA*, fractional anisotropy; *ACE-R*, Addenbrooke’s Cognitive Examination Revised

### Predictors of Cavitation

#### Clinical

There were no associations or correlations between the *cavitation index* and any of the following: age, sex, baseline clinical characteristics (NIHSS, modified Rankin Scale score, vascular risk factors), or conventional MRI parameters such as RSSI size and location, or concomitant CSVD burden, indicated by WMH severity, lacunes, PVS, microbleeds, or brain atrophy (Tables 1 and 2).

#### DTI

**Fig. 4:**
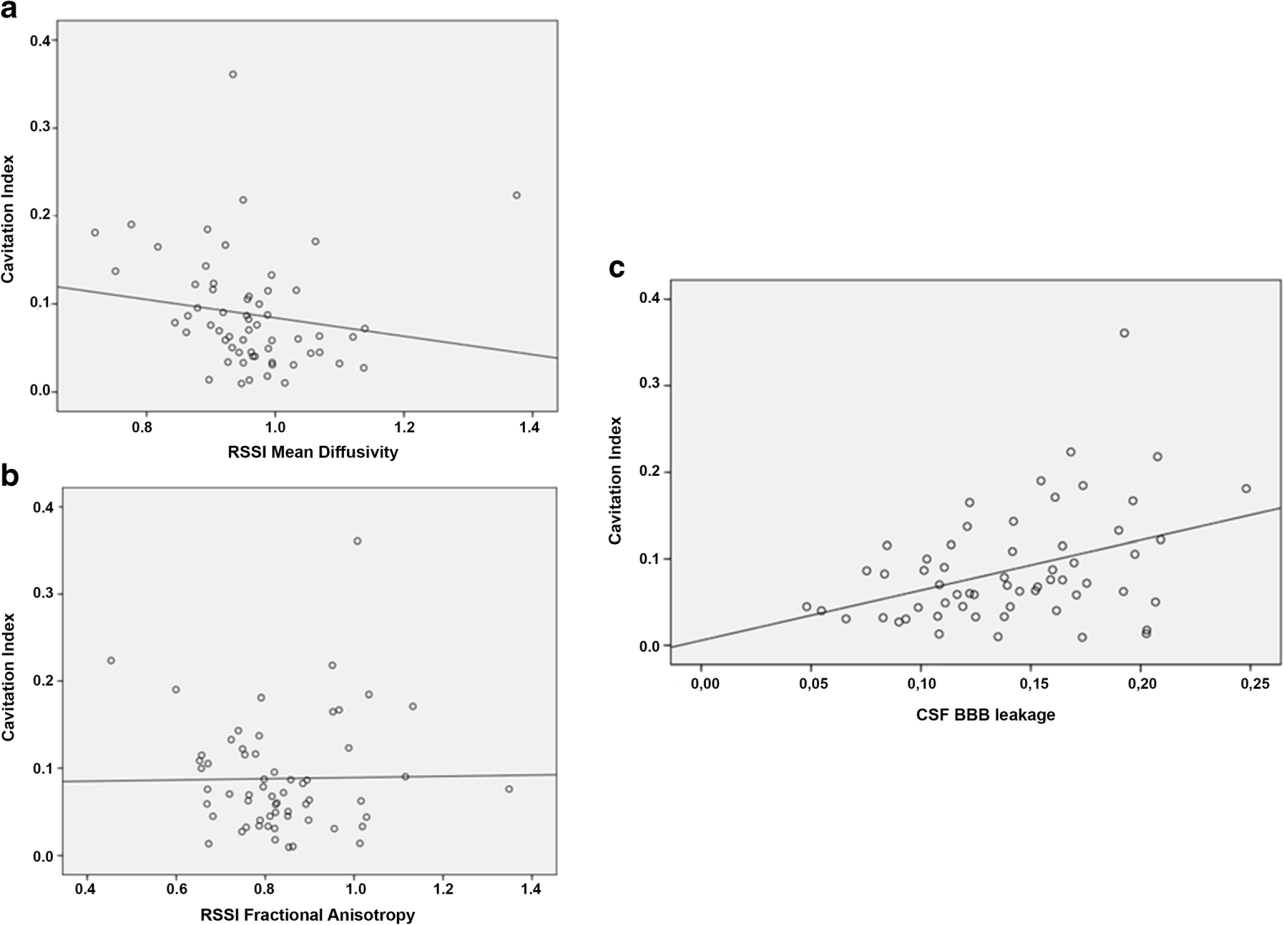
Correlation of the cavitation index with RSSI mean diffusivity (**a**) and fractional anisotropy (**b**), and BBB leakage in the CSF (normalized AUC of the signal enhancement curve on dynamic contrast-enhanced MRI, **c**)

At baseline, median FA and MD levels in normal-appearing white matter did not correlate with the *cavitation index*. Median FA and MD levels of the RSSI on baseline MRI were lower compared with the contralateral control region (FA: RSSI 0.35 vs. contralateral 0.42, *p* < 0.001; MD: RSSI 0.73 vs. contralateral 0.77, *p* < 0.001). After correction for the contralateral control region, baseline MD values of the RSSI were negatively correlated (*r*_s_ = − 0.371, *p* = 0.004) with the *cavitation index* but there was no correlation between corrected median FA values of the RSSI and cavitation at 1 year (*r*_s_ = − 0.084, *p* = 0.52; Table 2, Fig. 4). In a multivariable linear regression analysis controlling for age, the time period between symptom onset to baseline MRI, WMH severity, and RSSI size on DWI, the median MD value of the RSSI no longer significantly predicted the *cavitation index* (beta = − 0.235, *p* = 0.092).

#### BBB Leak

There were no correlations between BBB leakage in different tissues at 1–3-month post-stroke and the *cavitation index* at 1 year, except for BBB leakage in the CSF, which was associated with a higher *cavitation index* (*r*_s_ = 0.347, *p* = 0.007; Table 2, Fig. 4). In a multivariable linear regression analysis (controlling for age, the time period between symptom onset and DCE MRI at 1–3 months, WMH severity, and RSSI size), increased CSF BBB leakage remained independently positively associated with *cavitation index* at 1 year (beta = 0.315, *p* = 0.046).

## Discussion

In this study, nearly all patients with a symptomatic RSSI had some degree of cavity formation (median amount was 7%, ranging up to 36% of the original RSSI volume) on high-resolution, isotropic T1 MRI sequences 1 year after the index stroke event. To rate the amount of tissue destruction, we propose the RSSI *cavitation index* as a quantitative tissue outcome parameter, which we defined as the ratio of T1 cavity volume at 1-year post-stroke in relation to the RSSI volume on FLAIR-weighted scans at baseline. While lower MD levels of the RSSI at baseline MRI (indicating worse initial ischemia) and BBB leakage in the CSF at 1–3-month post-stroke correlated with a higher *cavitation index*, only the latter remained independently associated with the *cavitation index* in multivariable analyses.

Determining predictors for cavitation and the degree of tissue destruction following an RSSI is important for several reasons. These include identifying possible differences in tissue vulnerability and repair and a possible underestimation of brain tissue loss from previous RSSIs on cross-sectional imaging. Most importantly, the cavity formation after an RSSI is likely of clinical importance since the volume and number of lacunes are associated with cognitive decline in patients with CSVD [25]. In the present study, severity of cavitation had no effect on functional neurological or general cognitive outcome at 1-year post-stroke, but notably our sample was likely not large enough to comprehensively assess such associations.

In line with most previous studies, we did not find any associations between baseline demographic, clinical, or conventional MRI features of CSVD with subsequent cavitation [3, 5–7]. In contrast, another study from our center on a different cohort using CT or MRI to detect cavitation found deep brain atrophy was associated with cavitation, and diabetes and hypertension were more frequent in patients with non-cavitating lesions [4].

The present study suggests that the severity of cavitation is related to the degree of ischemia in the acute phase of the infarct since lower MD values of the RSSI (corrected for contralateral control region) correlated with a higher *cavitation index*. This finding agrees with a rodent stroke model and a previous study in patients with territorial ischemia [26, 27]. Likewise, a lower apparent diffusion coefficient (ADC) of the RSSI has been associated with subsequent RSSI cavitation (versus no cavitation) in another cohort of patients with lacunar infarction [3]. However, in the present study, the MD value of the RSSI was no longer predictive of cavitation severity after multivariable analysis. This is again consistent with the already mentioned study [3] and another investigation [28] using the ADC to estimate the initial degree of ischemia—both reports also showed that ADC levels of acute stroke lesions did not predict subsequent tissue outcome after adjustment for covariates. Moreover, we found no correlation of lesional FA values with cavitation. This agrees with previous work showing that FA is less predictive of acute stroke tissue fate [29] and could be explained by rather heterogeneous FA patterns in acute cerebral ischemia [30].

Most interestingly, we found that CSF BBB leakage at 1–3-month follow-up after stroke correlated with the *cavitation index* independently. While the present study was small, this finding supports the role of diffuse widespread endothelial dysfunction in the pathophysiology of CSVD [31]. In longitudinal studies, BBB leakage predicted recurrent stroke, more accumulating CSVD changes (progression of WMH), and cognitive decline in patients with lacunar stroke [11, 13]. Subtle BBB disruption could increase leakage of fibrinogen, which is toxic in interstitial tissues, increasing perivascular inflammation/immune activation and inducing neurodegeneration and progressive CSVD-related brain damage [13, 32]. The finding of increased levels of the neurofilament light chain protein (NfL) in the serum of RSSI patients with subsequent lesion cavitation could support this assumption [3]. While such an increase most likely comes from more extensive neuroaxonal damage [33], a leaky BBB could also facilitate the release of NfL into the blood. However, we also have to note that BBB leakage in other cerebral tissues was not associated with cavitation. This could potentially also be explained by the applied method, which might not be sensitive enough to detect more subtle BBB leakage in different parenchymal brain tissues.

In previous neuroimaging studies, there is heterogeneity regarding the morphological evolution of an RSSI. Depending on the (1) definition of a lacune/cavity, (2) applied neuroimaging modalities (CT or MRI) and MRI sequences, and (3) considered follow-up time periods, the rate of cavitation has been reported within a wide range between 48 and 94% [3–7]. Our study is in line with two previous reports showing that the incorporation of high-resolution T1 sequences significantly increases the sensitivity of detecting (often small) RSSI cavitation [3, 5]. While evidence for lacunar cavitation was present in nearly three-fourths on FLAIR and 85% on T2, almost all patients (93%) had a visible cavity on high-resolution T1, a number, which corresponds well with the study from Moreau and colleagues reporting cavitation on T1 sequences in 94% of patients at 90 days after the index infarct [5]. When we used co-registration and lesion magnification and neglected the widely used minimal lacunar diameter of 3 mm [2], we noticed that all patients had at least a small T1 hypointense lesion at 1-year follow-up MRI. Hence, the *cavitation index* was introduced to quantify the amount of complete tissue destruction. Previous studies only considered any cavitation versus non-cavitation but not the actual amount of tissue damage. Besides statistical advantages, our approach offers the possibility of quantifying the degree of cavitation in relation to the index stroke lesion. The *cavitation index* is similar to the “black hole ratio” used in multiple sclerosis to assess rates of tissue destruction and ongoing disease activity [23, 34, 35]. Our study also benefitted from the prospective recruitment of consecutive stroke patients, a thorough clinical workup and uniform, pre-specified MRI protocols at given time points on the same scanner.

We also have to consider several limitations. First, sample size was moderate, which potentially also limits the explanatory power of multivariable analyses. On the other hand, this is the largest study that has analyzed tissue outcome on RSSI in detail. Although MRI ratings have been performed without knowledge of clinical data and most MRI features were available from prior analyses, RSSI fate was rated by one single rater. However, inter-rater reliability measurements between two observers with different experiences and different methods revealed consistent results regarding cavitation volume—the main tissue outcome parameter of this study. Of note, T1 lesions were often small and therefore difficult to distinguish from enlarged perivascular spaces. However, while it is possible that some cavities represent residua of a perivascular space, we took particular care to avoid such misclassification with reference to baseline images. Moreover, exclusion of the four patients without an obvious T1 cavity did not significantly change the overall results. Irrespective of that, most of the RSSI tissue does not develop actual cavitation on T1 but remains hyperintense (or regains normal signal) on FLAIR and T2 sequences [36]. In future analyses, we will analyze DTI and BBB parameters in RSSI subregions that do or do not cavitate—an approach, which may help to understand the pathological processes in CSVD, tissue vulnerability, and repair. Finally, future studies should also examine in detail the clinical and especially cognitive domain-specific (long-term) consequences of more severe tissue destruction after an RSSI in larger cohorts.

## Electronic Supplementary Material

ESM 1 (PDF 105 kb)
