# Supplementary material for: Predictors of Lesion Cavitation After Recent Small Subcortical Stroke
Source: Transl Stroke Res. 2019 Nov 8;11(3):402–11. doi: 10.1007/s12975-019-00741-8 (PMC7235062; doi:10.1007/s12975-019-00741-8)
Supplement: Supplementary file 1 — (PDF 105 kb) [file 12975_2019_741_MOESM1_ESM.pdf]

Translational Stroke Research 2019: Supplementary Material

Predictors of lesion cavitation after recent small subcortical stroke

Running title: Predictors of lacunar cavitation

Thomas Gattringer (MD, PhD)[1,2], Maria Valdes Hernandez (PhD)[2], Anna Heye (PhD)[2], Paul A Armitage (PhD)[3], Stephen Makin (MD)[2,4], Francesca Chappell (MSc)[2], Daniela Pinter (PhD)[1], Fergus Doubal (MD)[2], Christian Enzinger (MD)[1], Franz Fazekas (MD)[1] and Joanna M Wardlaw (MD)[2]

1 Department of Neurology, Medical University of Graz, Austria

2 Centre for Clinical Brain Sciences, and UK Dementia Research Institute at the University of Edinburgh, Edinburgh, UK

3 Department of Infection, Immunity and Cardiovascular Disease, University of Sheffield, UK

4 Academic Section of Geriatric Medicine, Institute of Cardiovascular and Medical Sciences, University of Glasgow, Glasgow, UK

Corresponding author: Professor Joanna M Wardlaw

Centre for Clinical Brain Sciences

Chancellor's Building, 49 Little France Crescent

Edinburgh EH16 4SB, UK.

Telephone: +44 (0) 131 537 2943, Email: joanna.wardlaw@ed.ac.uk

Translational Stroke Research 2019: Supplementary Material

Inter-observer reliability of the T1-weighted cavity at one year follow-up

To recreate a practical scenario, two observers with different levels of experience delineated the boundaries of the recent small subcortical infarct (RSSI) on the same structural imaging modalities (i.e. T1-weighted MRI sequences) but pre-processed differently as ***Table 1*** shows. Observer 1, an experienced neurologist, used the software Mango (http://ric.uthscsa.edu/mango/) to generate the reference binary masks, while Observer 2 used the Region of Interest Tool in Analyze 12.0 (https://analyzedirect.com/analyze-12-0/). To evaluate the validity of the results obtained and limits of agreement, inter-observer reliability was assessed using Bland Altman plots [1].

Table 1. Image processing details of the ROIs used in the inter-observer agreement analyses

	ROIs generated by **Observer 1** (stroke neurologist)	ROIs generated by **Observer 2** (image analyst)
MRI sequence(s) used in the delineation of the cavity at 1-year follow-up	Original T1-weighted guided by DWI, FLAIR and T2-weighted	Original T1-weighted, guided by DWI and neuro-radiological reports
Method for delineating the ROIs	Manual boundary delineation	Semi-automatic by thresholding combined with a region-growing algorithm
Software used for delineating the ROIs	Mango	Analyze 12.0
Criteria followed for delineating the ROIs	DWI appearance, FLAIR and T1 signal and neuroradiological knowledge	DWI appearance, FLAIR/T1W signal, connected-component analyses, symmetry with the contralateral hemisphere, neuroradiological assessment notes, and T2 signal.

Results of the inter-observer agreement analyses

The volumetric difference of the cavity volume at 1 year was 2% between observers 1 and 2 albeit differences in the software, image space and methods used.

Table 2. Results of the inter-observer agreement analysis. Observer 1 measurements were considered as reference.

Parameters	Mean difference [95%CI]	% mean difference in average measure (SD)
1-year follow-up cavity volume	-2.25 [-39.85 35.35] mm^3	-2.40 (20.09) %

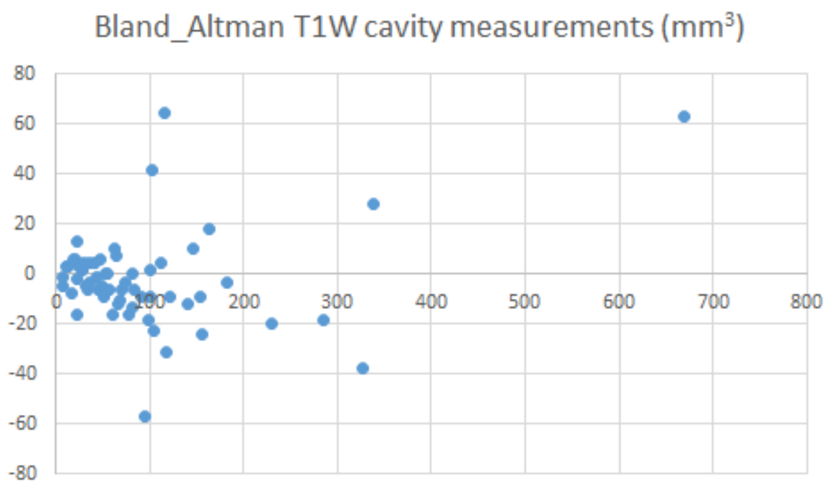

Figure 1. Bland-Altman analyses of the inter-observer volumetric differences in the delineation of the T1 cavitation at follow-up. Volumes (i.e. in both axes) are in mm^3.

References

[1] Bland JM, Altman DG. Statistical methods for assessing agreement between two methods of clinical measurement. Lancet. 1986 Feb 8;1(8476):307-10.
